# BRAND: Brand recognition and attitude norms database

**DOI:** 10.3758/s13428-024-02525-x

**Published:** 2024-12-16

**Authors:** Carolina Raffaelli, Elena Bocchi, Zachary Estes, James S. Adelman

**Affiliations:** 1 University of California San Diego, La Jolla, USA; 2 City University of London, London, UK; 3 Bocconi University, Milan, Italy; 4 University of Warwick, Coventry, UK

**Keywords:** Brand attitudes, Brand awareness, Brand logos, Brand recognition, Consumer responses

## Abstract

Research involving brands has increased substantially in recent decades. However, no extensive and free dataset of consumer responses to branding stimuli exists. The present research develops and validates such a dataset, which we call the *Brand Recognition and Attitude Norms Database* (*BRAND*). BRAND is the most comprehensive set of methodologically transparent, freely available, research-relevant consumer responses to branding stimuli, with measures of familiarity (awareness), liking (attitudes), and memory (recognition) of more than 500 top brands and their logos, spanning 32 industries. BRAND includes 5,356 primary datapoints aggregated from 244,400 raw datapoints (i.e., individual familiarity, liking, and memory responses) collected from 2000 US-resident consumers in 2 years (i.e., 2020 and 2024). The data exhibit good reliability, face validity, external validity, robustness across samples and time, cross-validity, and discriminant validity. BRAND can be broadly useful for testing hypotheses involving responses to brands, and for selecting stimuli in any study involving brands or logos. Thus, BRAND can facilitate research not only in consumer behavior and psychology but also in several related academic disciplines (e.g., economics, management, marketing).

## Introduction

Brands are ubiquitous and highly differentiated. They dominate markets ranging from the most basic goods (e.g., Charmin toilet paper) to the most advanced services (e.g., Goldman Sachs investments), and they differentiate along many dimensions of positioning, from price (e.g., economy versus premium; Geyskens et al., 2010) to personality (e.g., sincere versus exciting; Aaker, 1997). Consumers, in turn, purchase brands for many reasons and use them in myriad ways. For instance, some consumers purchase brands out of functional necessity, whereas others buy brands for hedonic pleasure (Hirschman & Holbrook, 1982; Voss et al., 2003). Moreover, some consumers use brands to develop their own identity (McCracken, 1986), whereas others use brands to signal their identity to others (Han et al., 2010). There is simply no denying that brands are prevalent and important in contemporary life and culture. With the prominence of brands in modern societies increasing dramatically, so has scholarly research on brands increased substantially in recent years.

Aside from the brand's name, the next most important brand element is the logo, which is a visual symbol representing the brand. A brand’s logo is an extremely important brand element because it affects brand attitudes (Janiszewski & Meyvis, 2001). The visual properties of logos, such as whether it is complete or incomplete (Hagtvedt, 2011), framed or open (Fajardo et al., 2016), static or dynamic (Cian et al., 2014), angular or circular (Jiang et al., 2016), symmetric or asymmetric (Luffarelli et al., 2019a, 2019b), can affect consumers’ inferences about and evaluations of the brand. In fact, logos can also impact brand authenticity and commitment and, ultimately, the brand’s financial performance (Luffarelli et al., 2019a, 2019b; Park et al., 2013). Like branding more generally, scholarly research on logos has also increased substantially.

Given the ubiquity and importance of brands and logos, and given the growth in research on them, we find it surprising that there is no extensive and freely available dataset of consumer responses to brands and logos. There is no comprehensive dataset that researchers can use to test whether some property generally predicts brand attitudes. Nor is there any dataset to assist researchers in selecting brands or logos that are matched on some properties (e.g., familiarity) and vary on others (e.g., memorability). The present research develops and validates such a dataset, which we call the *Brand Recognition and Attitude Norms Database*, or *BRAND*. Before describing the dataset, we first review the current state of research on brands and logos, in order to identify how BRAND can facilitate research in consumer behavior, economics, marketing, and psychology.

## Branding research: State of the art

Some of the most important research on branding has been purely theoretical (Keller, 1993) or qualitative (Brown et al., 2003). Such studies can provide insightful overviews or an in-depth understanding of consumer responses to brands. The present research, however, focuses on quantitative studies of branding that use regression or experiment designs. In this section, we briefly review how quantitative research on brands and logos is typically conducted, and the datasets that are available to facilitate such research.

### Empirical approaches

Just as in other areas of research, quantitative studies of branding usually take one of two basic approaches. One approach, which we call *regression-generalization studies*, is to demonstrate that some factor of interest predicts some brand-related outcome (e.g., brand attitudes; sales). Such studies typically use regression analyses to generalize an effect across a large number of branding stimuli, while statistically accounting for other relevant factors. Regression-generalization studies are common in brand-based research. For example, in their seminal investigation of consumer responses to logos, Henderson and Cote (1998) measured a broad range of design attributes (e.g., naturalness, elaborateness) for 195 logos. They then conducted regression analyses to identify which of those factors best predicted consumers’ attitudes toward and memory of the logos.

A second approach to branding research, which we refer to as *experimental-proof studies*, is to demonstrate that, under some conditions at least, some factor of interest affects some response to branding stimuli (e.g., logo memory; purchase intentions). Essentially, such studies employ experimental designs to provide an existence proof that some property of brands can affect some attitude or behavior, while methodologically controlling other relevant factors. Experimental-proof studies are extremely common in brand-based research, and they typically include only one or a few brands. For instance, in a field experiment, Walsh et al. (2010) used Adidas and New Balance brands to investigate the effect of logo design on brand attitudes.

### Extant datasets

Several lists of “top brands” are produced regularly, with some limited amount of data freely available in them. We summarize these brand lists in Table 1. Perhaps the most famous is *Interbrand*’s “Best Global Brands” list, and *Forbes*, *Kantar* (BrandZ), *Tenet*, and *Brand Finance* produce similar lists. Most of these lists include not only brand rankings but also some proprietary measure of “brand value,” which is based largely on financial performance (e.g., revenues) but which typically also incorporates an estimate of brand equity (e.g., consumer attitudes). See the Appendix for descriptions of these brand value calculations.

**Table 1:** Extant brand datasets

Source	Report	Brands (*N*)	Key measures
Brand Finance	US 500	500	Rank, value
BrandZ (Kantar)	Top 100 Most Valuable US Brands	100	Rank, value, contribution
Forbes	World's Most Valuable Brands	100	Rank, value, revenues
Interbrand	Best Global Brands	100	Rank, value
Tenet	Top 100 Most Powerful Brands	100	Rank

Researchers often use these existing datasets to select brands for use in their studies or to test hypotheses about brands. For instance, Labrecque and Milne (2012) and Luffarelli et al., (2019a, 2019b) utilized the Interbrand list to study the relationship between logo characteristics and brand personality. Van der Lans et al. (2014) examined brand alliances using brands from the Interbrand and BrandZ lists. Johansson et al. (2012) and Madden et al. (2006) used Interbrand brands to represent high-equity brands, and investigated the impact of brand equity on stock returns, respectively. Chu and Keh (2006) explored strategic investments' effects on brand value with the Interbrand list, while Harjoto and Salas (2017) and Gherghina and Simionescu (2015) investigated corporate social responsibility and brand value using Interbrand and Brand Finance values, respectively. Similar examples abound.

Although the extant datasets have proven useful for some research purposes, they also suffer several critical limitations. First, extant datasets tend to include relatively few brands. Most brand datasets, such as Interbrand’s list, include only 100 brands. Those lists thus provide little statistical power for testing hypotheses about brands. Second, because such datasets are proprietary, they tend to provide relatively little detail about the methods used to sample the brands, to generate the data, and/or calculate the brand values. Thus, due to a lack of transparency, academic researchers are generally unable to assess the extent to which the data are suitable for their purposes, much less whether they are reliable and valid. Third, although most brand lists do include some more or less direct measure of brand equity (i.e., consumer perceptions and/or opinions), the currently available brand datasets are based mostly on financial performance in terms of sales, revenues, and so on. Those datasets lack some measures that are of great interest to academic researchers, such as brand awareness, attitudes, and recognition. Fourth, although some more consumer-focused brand lists are available from branding consultancies, they tend to be costly. For instance, the BrandAsset Valuator (BAV) includes equity measures such as brand knowledge, brand personality, and usage frequency, but those data are sold at costs on the magnitude of tens of thousands of dollars, which is beyond the reach of most academic research budgets. A final limitation of extant datasets is that they only include evaluations of the brand. There are currently no rankings or evaluations of other influential brand elements, such as logos.

In sum, brand-based research has increased dramatically in recent years, utilizing both the regression-generalization and the experimental-proof approaches. As explained below, a large dataset of consumer responses to brands could benefit both of those empirical approaches to brand-based research. Although a few datasets of brand information do exist, they include relatively few brands, their evaluation methodologies tend to be opaque, they lack measures that are of interest to many academic researchers, and those that do include measures of consumer equity tend to be financially inaccessible to academic researchers, and they lack different brand elements such as logos. The BRAND database addresses those limitations.

#### BRAND

The present research aims to generate and validate an extensive set of freely available, methodologically transparent, research-relevant consumer responses to brands. Specifically, we created a dataset of the familiarity, liking, and memorability of more than 500 top brands and their logos. We refer to this dataset as the *Brand Recognition and Attitude Norms Database*, or *BRAND*. We collected BRAND data initially in 2020 (i.e., *BRAND 2020*) and again in 2024 (i.e., *BRAND 2024*).

### What is it?

BRAND is the most comprehensive dataset of frequently researched consumer responses to branding stimuli. As shown in Table 1, the *Brand Finance* list of top brands is the most extensive freely available list, including 500 brands. We therefore based our BRAND database on this Brand Finance list. Although the Brand Finance list is nominally focused on “US brands,” it nonetheless includes many top global brands. For instance, 57 of the 100 brands on the Forbes (2020) “World’s Most Valuable Brands” list also appeared in the Brand Finance (2019) list, and hence in BRAND 2020. In addition to the brands themselves, we also collected consumer responses to the brands’ logos, because logos critically affect brands’ financial performance (Luffarelli et al., 2019a, 2019b; Park et al., 2013) and because they are a common topic of research in their own right. Thus, BRAND 2020 includes 500 top brands and their logos, for a total of 1000 stimuli.

Researchers often study brand awareness, brand attitudes, and brand recognition, as these measures are key indicators of brand equity (Keller, 1993). For instance, Kent and Allen (1994) and Campbell and Keller (2003) investigated the effects of brand awareness on advertising effectiveness, Percy and Rossiter (1992) examined the relationship between brand awareness and brand attitudes in the context of advertising, and Keller (1987) assessed the effects of advertising on brand attitudes and brand recognition. We therefore measured those three consumer responses in our dataset. We assessed awareness via a measure of familiarity, attitudes via a measure of liking, and recognition via a memory test.

BRAND 2024 is an update and extension of BRAND 2020. It includes all 492 surviving brands from BRAND 2020, that is, the 500 original brands minus eight brands that ceased operating before 2024 (e.g., Twitter). It also includes 97 new entrants to the Brand Finance list in 2023 (e.g., X). BRAND 2024 includes familiarity and liking ratings, but not memory. BRAND 2024 therefore consists of the familiarity and liking of 589 brands and their logos. Overall then, the BRAND dataset includes 597 brands: 500 from BRAND 2020, and 97 that appear in BRAND 2024 but not in BRAND 2020.

Our data collection procedures closely followed those typically employed in cognitive psychology and psycholinguistics, where open sharing of large datasets of attitudinal and behavioral responses to words (cf. brands) and images (cf. logos) is common (e.g., Andrade et al., 2023; Balota et al., 2007; Dan-Glauser & Scherer, 2011; Muraki et al., 2023; Stoinski et al., 2023; Winter et al., 2024).

### What is it for?

BRAND has two primary uses: hypothesis testing and stimulus selection. BRAND provides a standardized basis for hypothesis testing via the regression-generalization approach. In fact, BRAND can be useful for retrieving dependent variables, predictor variables, and/or control variables. Regarding dependent variables, any researcher who wishes to test whether some factor predicts recognition of, attitudes toward, or memorability of brands or logos has simply to merge a measure of the given factor with the measures in BRAND. For example, if one hypothesizes that consumers like brand names with a certain phonetic property (e.g., Lowrey & Shrum, 2007), one need only code whether each of the brand names in BRAND has that phonetic property and then use those coded values to predict brand attitudes within BRAND (see Study 2). If one hypothesizes that circular logos are liked more than square logos (e.g., Jiang et al., 2016), then they can simply collect ratings of how rounded or squared the BRAND logos are and test whether those ratings predict consumer attitudes toward the logos in BRAND.

Regarding predictor variables, BRAND can also be used to test whether recognition of, attitudes toward, or memorability of brands or logos predicts some other outcome. Want to know whether logo attitudes predict sales (e.g., Park et al., 2013)? Simply retrieve attitudes toward the logos in BRAND, retrieve those brands’ financial performance data from Compustat, merge the two datasets, and run a regression. Want to know whether well-known logos counterintuitively elicit worse memory (cf. Blake et al., 2015)? Retrieve logo familiarity ratings and logo memory scores from BRAND to find out (see Study 1).

BRAND can also be useful for control variables. Suppose a researcher investigates whether the introduction of a new brand extension increases sales of the brand’s other offerings (Balachander & Ghose, 2003; Swaminathan et al., 2001). Given that some brands – but not others – introduce a new brand extension during the period of study, the researcher could retrieve brand attitudes from BRAND as a control factor, in order to ensure that the effect of a brand extension on sales is not simply due to a confound of highly liked brands being more likely to introduce brand extensions. Alternatively, the researcher could test whether brand attitudes moderate the presumed effect.

Second, BRAND provides a tool for stimulus selection in experimental-proof studies. In fact, just as in regression-generalization studies, BRAND can be useful for both manipulations and controls here also in experimental-proof studies. In terms of manipulations, for instance, when investigating product choices between famous brands and lesser-known brands (e.g., Thoma & Williams, 2013), the familiarity ratings in BRAND could be used to identify more and less known brands. To investigate how brand attitude affects word of mouth, one may consult BRAND to identify brands that vary in liking but are matched on familiarity and memorability. In terms of controls, BRAND may be even more broadly useful. If an experimenter investigates the influence of repetition on liking and memory of logos (Janiszewski & Meyvis, 2001; Van Grinsven & Das, 2016), they could consult BRAND to identify logos that are matched on familiarity, liking, and memorability outside the context of the experimental repetitions. If one wishes to test whether advertisements with celebrity endorsers distract attention away from the brand (Erfgen et al., 2015), they could use BRAND to identify brands that are matched for familiarity and liking, so that any difference in brand memory would be attributable to the presence or absence of a celebrity endorser, rather than to familiarity or liking of the brands.

Thus, whether used as dependent variables (e.g., logo memorability), predictor variables (e.g., brand attitude), or control variables (e.g., brand familiarity), BRAND is ideal for hypothesis testing via regression-generalization studies. BRAND is also ideal for stimulus selection in experimental-proof studies, allowing researchers to easily identify brands or logos that are matched on some consumer responses (e.g., familiarity) and/or that vary on other consumer responses (e.g., attitudes).

### Who is it for?

Most obviously, BRAND is useful for branding researchers. Any researcher who studies brands and/or logos can benefit from using BRAND to test hypotheses about awareness, attitudes, and/or recognition, to control for those consumer responses when testing hypotheses about other behaviors or outcomes, and to select well-controlled stimuli in experimental-proof studies of brands and logos.

BRAND may also benefit other marketing researchers who do not specifically investigate branding. Essentially *any* researcher who uses brands or logos as stimuli can benefit. For instance, advertising researchers can use BRAND to identify suitable brands (e.g., moderate attitudes) to use in their ads. A social media researcher who investigates the impact of corporate tweets on earned media impressions can retrieve brand attitudes from BRAND and include them as a control factor in regression analyses. A marketing strategy scholar who studies the effect of new product launches on revenues can use BRAND to retrieve consumers’ prior attitudes toward the given brands as a potential moderator of the effect. And so on.

BRAND can also benefit researchers in psychology. For instance, academic psychologists sometimes use branding stimuli to test theoretical predictions and/or validate findings with more naturalistic stimuli. Buttle and Westoby (2006) used brand logos (e.g., Apple, Camel, Nike) to investigate the phenomenon of repetition blindness in visual perception, and Streicher and Estes (2015) used Coca-Cola bottles and Red Bull cans to demonstrate that tactile processing facilitates visual perception. Khan et al. (2013) found that more conservative people were more likely to prefer established national brands, and Walasek and Brown (2015) showed that status goods, such as designer brands (e.g., Ralph Lauren), are searched more frequently in US states with high income inequality than in more equal states.

BRAND may also prove useful for researchers in several other related fields, including not only similar disciplines such as management (e.g., Wood, 2000) and economics (e.g., Head & Mayer, 2019) but also less similar disciplines such as sociology (e.g., Hamilton, 2012) and cultural anthropology (e.g., Foster, 2007). Thus, BRAND can facilitate research both within and beyond consumer behavior and psychology.

## Overview of studies

We generated and validated BRAND across three studies. Study 1 generated and validated BRAND 2020. Conducted in 2020 on the Brand Finance (2019) US 500 list, this study included measures of the familiarity, liking, and memorability of 500 top brands and their logos. Analyses established the validity and reliability of the dataset in several ways. Study 2 then tested the external validity and utility of BRAND by replicating and generalizing a prior result from the branding literature. Specifically, using fictitious brand names, Lowrey and Shrum (2007) found that consumers like brand names with back vowels (e.g., “Nallen”) more than those with front vowels (e.g., “Nillen”). Study 2 reveals that this effect also holds across a much larger set of real brands in BRAND 2020, demonstrating the validity and utility of BRAND. Finally, Study 3 generated and validated BRAND 2024. Conducted in 2024 on the Brand Finance (2023) US 500 list, this study included measures of the familiarity and liking of 589 brands and their logos. BRAND 2024 includes all surviving brands and logos from BRAND 2020 (i.e., brands that ceased trading between 2020 and 2024 were removed), plus all new entrants on the 2023 Brand Finance list (i.e., brands that appeared in Brand Finance’s 2023 list but not on their 2019 list). Thus, the comprehensive BRAND dataset, integrating both 2020 and 2024, includes a total of 597 brands and logos. This encompasses the initial 500 from BRAND 2020, supplemented by an additional 97 brands that were introduced in BRAND 2024 and were not present in 2020. All stimuli, all surveys, and all data^1^ are freely available at https://researchbox.org/1892.

## Study 1: BRAND 2020

BRAND 2020 includes measures of familiarity, attitudes, and memorability of 500 top brands and their logos, for a total of 3000 aggregated datapoints (i.e., 500 brands and 500 logos × 3 measures). Those 3000 datapoints are aggregated from approximately 150,000 responses from approximately 1200 US-resident consumers. Each respondent evaluated 50–100 brands or logos, and each datapoint is aggregated across approximately 30–60 consumer responses.

### Methods

#### Participants

One thousand two hundred six respondents from the Prolific online research panel participated for pay. Respondents’ ages ranged from 18 to 81, with a mean of 32 years (SD = 12); 54.0% self-identified as female, 43.6% as male, and 2.4% chose not to say. We used a screening function in Prolific to restrict eligibility to users who reported current residence in the U.S., and we subsequently retrieved demographic data from Prolific confirming that nearly all participants (94.4%) self-reported residence in the US (“prefer not to say” = 5.6%). We excluded two participants who reported residence outside the US.

Data were collected from four independent groups. Of the 1204 US-resident participants, approximately half (*N* = 605) rated their familiarity with and liking of the stimuli (henceforth *familiarity-liking* group), and after excluding five participants who failed an attention check (see *Procedure*), 600 valid participants remained in this group. The other half of the participants (*N* = 599) instead rated their liking and then completed a memory test for the stimuli (*liking-memory* group), and after excluding five participants who reported a problem with the presentation of logos (see *Procedure*) and two participants who completed the survey twice, 592 valid participants remained in this group. Within each of those two groups, each participant judged either the brands or their logos, but not both. Of the 600 valid participants in the familiarity-liking group, approximately half rated the brands (*N* = 297) and half rated the logos (*N* = 303). Furthermore, of the 592 valid participants in the liking-memory group, approximately half rated the brands (*N* = 293) and half rated the logos (*N* = 299). Table 2 summarizes the basic demographic characteristics of the respondents in each group.^2^

**Table 2:** Respondent characteristics (after exclusions) in BRAND 2020

			Age				Gender	
Group	Stimuli	*N*	Min	Max	M	SD	Female	Male
Familiarity-Liking	Brands	297	18	79	30.57	9.98	61%	39%
	Logos	303	18	67	30.46	10.69	61%	39%
Liking-Memory	Brands	293	18	81	32.83	13.26	50%	50%
	Logos	299	18	78	34.90	11.94	49%	51%
	Overall	1192	18	81	32.19	11.47	55%	45%

2.4% of respondents who chose not to report their age and 2.52% who chose not to report their gender are excluded from those respective statistics in the table

#### Stimuli

We selected the Brand Finance (2019) US 500 because it was the most extensive list of “top brands,” with a total of 500 brands spanning 32 industries, from aerospace to utilities (see Table 3).^3^ This Brand Finance list uses the “Royalty Relief” method of brand valuation, which is defined as the “net economic benefit that a licensor would achieve by licensing the brand in the open market” (Brand Finance, 2019, p. 30). That is, *brand value* is derived not only from the brand’s financial performance but also from its brand equity (i.e., consumers’ subjective valuation of the brand). For instance, in the apparel category, Victoria’s Secret and Old Navy generated approximately equal revenues in 2019, but Victoria’s Secret had a far higher brand value due to consumers’ more positive perceptions of the brand.

**Table 3:** Brand characteristics in Brand Finance, 2019 and 2023

		Brand Finance 2019 Rank			Brand Finance 2023 Rank		
Industry	Example	*N*	M	SD	*N*	M	SD
Aerospace & Defense	Boeing	10	209	155	8	197	111
Airlines	Delta	6	194	164	4	126	50
Apparel	Nike	17	260	130	14	279	124
Auto Components	Lear Corp	0	-	-	1	445	-
Automobiles	Ford	11	249	146	11	212	135
Banking	Wells Fargo	31	218	147	32	194	136
Beers	Budweiser	5	262	146	5	292	146
Car Rental Services	Enterprise	3	282	150	5	310	136
Chemicals	Dow	5	291	132	5	329	102
Commercial Services	Deloitte	27	266	159	32	257	150
Cosmetics & Personal Care	Johnson's	27	258	126	19	261	98
Engineering & Construction	General Electric	22	258	114	22	263	125
Exchanges	CME	2	448	74	3	454	54
Food	Purina	24	322	132	30	330	138
Healthcare	UnitedHealthcare	27	257	150	27	219	140
Hotels	Hilton	11	300	124	7	244	116
Household Products	Scotts Miracle-Gro	0	-	-	1	454	-
Insurance	GEICO	15	240	152	19	263	162
IT Services	Accenture	9	199	153	5	268	202
Leisure & Tourism	Royal Caribbean	6	321	75	8	413	65
Logistics	UPS	8	175	152	12	235	164
Media	Disney	20	206	156	25	258	154
Oil & Gas	Chevron	26	286	139	25	286	129
Pharma	Pfizer	10	302	99	8	247	99
Real Estate	CBRE	4	408	56	2	370	60
Restaurants	Starbucks	13	189	129	17	209	147
Retail	Walmart	42	237	151	38	193	134
Soft Drinks	Coca-Cola	11	223	118	10	244	149
Spirits	Jack Daniel's	2	210	25	2	334	93
Tech	Amazon	70	216	154	71	213	152
Telecoms	AT&T	10	168	154	8	174	171
Tires	Dunlop	2	379	8	2	420	22
Tobacco	Marlboro	14	285	126	13	276	120
Utilities	Exelon	10	404	70	9	400	76

Because the Brand Finance report does not include the brand logos, we manually and extensively searched for and collected each brand’s logo. For every brand, we first retrieved the logo displayed on the US version of its corporate website. However, we observed that many brands use multiple logos, raising the question of which of them we should include in our dataset. For instance, Facebook uses different logos on its webpage (which includes the full name “Facebook”) and its mobile app (which includes only the letter “f”). Given the pragmatic constraint that we could include only one logo for each brand, we conducted a secondary search for alternative logos, and we developed a set of decision rules to identify a single logo for each brand that we would include in our dataset. This procedure was challenging and cumbersome, as described next.

For every brand, we searched for alternative logos that may appear on the product, in the retail channel (e.g., on packaging), in the app store, or in other points of contact with consumers. Most brands (60% of them) used the same logo consistently across those various consumer touchpoints. However, 200 brands (40%) used different logos in different touchpoints, as in the Facebook example above. After reviewing those discrepant cases, we established a set of rules to determine which logo to include in our dataset. Those rules, and the number of cases decided by each rule, are described in Table 4. After implementing seven rules summarized in the table, we were left with 42 “miscellaneous” cases that could not be determined by any of the other rules. In those cases, two of the authors independently judged which was the brand’s primary logo, based on another review of each brand’s various touchpoints. In case of disagreement, another author cast a tie-breaking judgment.

**Table 4:** Logo selection rules

		*N*		
	Selection Rule	2020	2024	Description
Consistent logo use		300	321	The brand uses the same logo consistently across various consumer touchpoints
Inconsistent logo use		200	277	The brand uses different logos across touchpoints
	App	11	15	If one version is the app icon, then take the version that is not the app icon
	Color	44	82	If the two versions display different colors, then take the one that is most prominent in the company's website
	Orientation	28	48	If the two versions of the logo are differently oriented, then take the one on the product or the mode on Google Images
	Movie	4	5	If one version is a stylized logo displayed in movies (i.e., for film-related brands), then take the stylized version
	Part	33	40	If one version is part of the other, then take the most complete version
	Slogan	30	31	If one version includes the slogan, then take the one without the slogan
	Restaurant	8	13	If one version is found on the restaurant signboard, then take the one on the signboard
	Miscellaneous	42	43	If none of the rules above applied, then the logo was selected by consensus among authors

Whenever possible, we retrieved the brand’s logo without a background, unless it was an integral part of the logo (e.g., the Goldman Sachs logo consists of the name inside a blue square). We resized all logos to have similar dimensions by positioning them at a minimum distance from the edges of a standardized frame, and we saved them as 400 × 225 resolution portable network graphic (png) files.

#### Procedure

This research was approved by the research ethics committee at the host university. After providing informed consent, participants in the familiarity-liking group read the following attention check: “How much attention are you willing and able to dedicate to this task? If you select VERY LITTLE, then we will have to reject your work” (options: very little; a whole lot). The data of all participants who selected “very little” were excluded from analyses (*N* = 5).

The study included 1000 stimuli in total (500 brands + 500 logos). To minimize fatigue and demotivation, each participant evaluated either 50 brands or 50 logos (between-participants). The 500 brands were randomly allocated among ten lists of 50 brands, and we similarly created ten lists of 50 logos. Each participant was randomly assigned to one of those lists, and the 50 stimuli within the assigned list were presented in random order.^4^

Participants were informed that “We are investigating consumer opinions about [brands/logos].” Participants assigned to evaluate logos were additionally informed that “A logo is a visual symbol representing a brand…**Please evaluate the logo (i.e., the visual symbol), rather than the brand that it represents.**” Participants in the familiarity-liking group rated the stimuli in terms of familiarity (“Please rate to what extent you are **familiar** with this [brand/logo];” 1 = *not familiar at all*; 7 = *very familiar*) and liking (“Please rate to what extent you **like or dislike** this [brand/logo];” 1 = *dislike*; 7 = *like*). Each stimulus was presented on a separate page, with both rating scales appearing below the brand name or logo.

Participants in the liking-memory group completed two experimental phases. In the *exposure phase*, participants read the same instructions but were additionally informed that the study would last approximately 10–12 min, and also read the following: “**Please do not start this study unless you are able to complete the entire task without a break. If you take a break in the middle, you may be timed out.**” Participants then rated their liking of 50 stimuli, exactly as in the familiarity-liking group, except without rating familiarity. Participants were not informed that there would be a second phase of the study. Upon completion of the exposure phase, however, participants immediately began a *test phase*, which consisted of a recognition memory test. They read the following interim instructions:

“You have completed the first part of the study. **Please now begin the second part of the study without taking a break. If you take a break, you may be timed out.** In this second (and final) part of the study, we will show you 100 [brands/logos]. Some of these [brands/logos] will be ones that you saw in the first part of the study (i.e., "old"), whereas others will be [brands/logos] that you did not see in the first part (i.e., "new"). **Please click 'old' if you think that you saw the [brand/logo] in the previous part of the study, or click 'new' if you think you didn't.**”

In addition to the 50 (old) stimuli that the participant evaluated in the exposure phase, participants were also shown 50 (new) stimuli from one of the other lists. Each list of 50 stimuli was yoked with another list of 50 stimuli, so that all stimuli appeared approximately equally often as an old stimulus and as a new stimulus. Each stimulus appeared on a separate page, above the “old” and “new” response options, and the 100 stimuli appeared in random order. The recognition memory task was modeled after Cortese et al. (2010).

Finally, at the end of the study, participants in the liking-memory group were asked an additional question. Because a few participants in the familiarity-liking group commented in a feedback textbox that sometimes the logos took a while to load or occasionally did not load at all, in this liking-memory task we added a question at the end of the study to assess whether this problem had occurred. Participants were asked “When you were doing the experiment, did all [logos/brands] clearly appear on your screen?” Response options were “Yes, all [logos/brands] clearly appeared” and “No, some [logos/brands] never appeared.” Participants who indicated that some logos or brands did not appear were then asked “How many [logos/brands] do you think you have missed?”, and they typed their estimate into a textbox. Only 14 participants (i.e., 2.4% of the sample) indicated that some stimuli did not appear, and those 14 participants’ mean estimate of the number of stimuli that did not appear was 9 (SD = 13).^5^ The data of all participants who reported missing ten or more stimuli were excluded from analyses (*N* = 5).

### Results

#### Data aggregation

In the BRAND dataset, the brand is the unit of analysis. For each measure of each brand (e.g., familiarity with the Nike logo), we aggregated across all participants’ responses. For continuous measures (i.e., familiarity and liking ratings), these were averages. For categorical measures (i.e., memory judgments), these were proportions. Each brand was rated for familiarity by approximately 30 participants (min = 29, max = 31, SD = 0.64). Because both groups of participants (i.e., familiarity-liking and liking-memory) rated their liking of the brands, each brand received about twice as many liking ratings: approximately 59 participants (min = 58, max = 60, SD = 0.78). And because each brand appeared as an “old” memory stimulus for some participants and as a “new” stimulus for others, each brand also received memory judgments by approximately 59 participants (min = 58, max = 60, SD = 0.80). Analogously, each logo received approximately 30 familiarity ratings (min = 28, max = 32, SD = 1.27), 60 liking ratings (min = 56, max = 63, SD = 2.48), and 60 memory judgments (min = 57, max = 62, SD = 1.72).^6^

#### Memory measures

We analyzed memory performance via signal detection theory, with four primary measures: A *hit* occurs when an “old” stimulus is correctly identified as old; a *miss* occurs when an old stimulus is incorrectly judged as new; a *false alarm* occurs when a new stimulus is incorrectly judged as old; and a *correct rejection* occurs when a new stimulus is correctly identified as new. One simple and common method for converting those primary measures into an accuracy score, which we refer to as *corrected accuracy* (Stanislaw & Todorov, 1999), is to subtract the false alarm rate from the hit rate (i.e., Hits–False Alarms). This score essentially reflects the likelihood of correctly recognizing an old stimulus, while also correcting for response bias (captured here by the false alarm rate). A similar but more formal measure, *d’*, subtracts the *z*-score of the false alarm rate from the *z*-score of the hit rate (i.e., *Z*_Hits_ – *Z*_False Alarms_), thus providing a standardized accuracy measure in SD units. We include all six of these memory measures in the BRAND 2020 dataset. However, because corrected accuracy and *d’* were extremely correlated (brands: *r* = 0.98, *p* < 0.001; logos: *r* = 0.97, *p* < 0.001), in all other text, tables, and figures, we report corrected accuracy (Hits–False Alarms) as our primary measure of recognition memory, and we do not discuss *d’* further.

#### Distributions

**Fig. 1:**
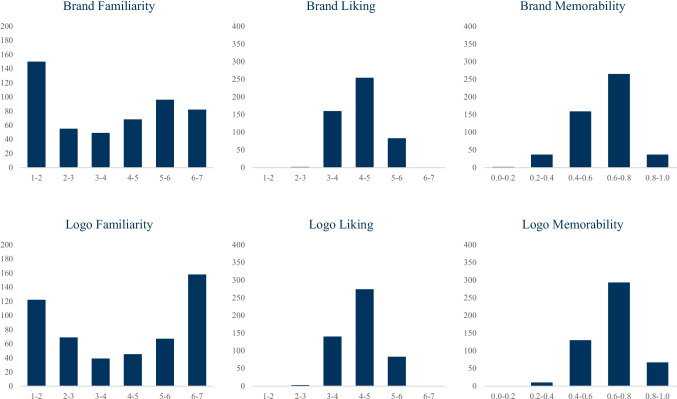
Distributions of the familiarity, liking, and memorability (corrected accuracy) of brands and their logos in BRAND 2020

Descriptive statistics of the familiarity ratings, liking ratings, and memory scores for the brands and logos are reported in Table 5 and histograms of the six focal measures are shown in Fig. 1. *Brand familiarity* ratings ranged from 1.00 (Parker-Hannifin, Lam Research) to 6.90 (Target), with a mean of 3.76 (SD = 1.92). Notably, however, the distribution is bimodal (Fig. 1). *Brand liking* ratings ranged from 2.22 (Marlboro) to 6.17 (Netflix), with a mean of 4.35 (SD = 0.57). As shown in Fig. 1, the distribution was normal. *Brand memorability* (corrected accuracy) scores ranged from 0.03 (ADT) to 0.90 (Olive Garden, Safeway, Dick’s Sporting Goods, Lincoln), with a mean of 0.62 (SD = 0.14), and were normally distributed. *Logo familiarity* ratings ranged from 1.00 (CA Technologies) to 6.97 (Amazon, Ford, Walmart), with a mean of 4.18 (SD = 2.09). As with brands, logo familiarity shows a bimodal distribution with peaks near 1 and 7 (Fig. 1). *Logo liking* ratings ranged from 2.72 (Arrow Electronics) to 5.83 (FedEx), with a mean of 4.39 (SD = 0.61), and were normally distributed. *Logo memorability* scores ranged from 0.24 (HCL) to 0.94 (GEICO), with a mean of 0.66 (SD = 0.12), and were normally distributed.

**Table 5:** Descriptive statistics of measured variables in BRAND 2020

Stimuli	Measure	*N*	Min	Max	M	SD	Skew
Brands	Familiarity	500	1.00	6.90	3.76	1.92	0.01
	Liking	500	2.22	6.17	4.35	0.57	0.48
	Hits	500	0.46	0.98	0.80	0.11	– 0.65
	Misses	500	0.02	0.54	0.20	0.11	0.65
	False Alarms	500	0.02	0.83	0.18	0.09	1.50
	Correct Rejections	500	0.17	0.98	0.82	0.09	– 1.50
	Corrected Accuracy	500	0.03	0.90	0.62	0.14	– 0.59
	d'	500	0.12	3.30	1.88	0.55	0.03
Logos	Familiarity	500	1.00	6.97	4.18	2.09	– 0.11
	Liking	500	2.72	5.83	4.39	0.61	0.00
	Hits	500	0.44	0.98	0.81	0.09	– 0.42
	Misses	500	0.02	0.56	0.19	0.09	0.42
	False Alarms	500	0.02	0.40	0.15	0.07	0.56
	Correct Rejections	500	0.60	0.98	0.85	0.07	– 0.56
	Corrected Accuracy	500	0.24	0.94	0.66	0.12	– 0.40
	d'	500	0.62	3.70	2.06	0.53	0.33

#### Reliability

Recall that two independent groups of participants rated their liking of the BRAND 2020 stimuli: Some participants rated familiarity and liking, whereas others rated liking and then completed a memory test. We therefore used those independent sets of liking ratings to assess test–retest reliability. Brand-liking ratings by the familiarity-liking group and the liking-memory group correlated strongly, *r* = 0.79, *p* < 0.001. Logo liking ratings by the two groups of participants also correlated strongly, *r* = 0.77, *p* < 0.001. Thus, the reliability of BRAND 2020 was good.

#### Correlations among measures

Table 6 displays the correlations among the measures of BRAND 2020 (see lines 1-6). As expected, the six measures all correlated positively with each other. Here, we highlight two observations that are theoretically and practically meaningful. First, more familiar brands were liked more (*r* = 0.73) than less familiar brands, and more familiar logos were also liked more (*r* = 0.72) than less familiar logos. These results corroborate the mere exposure effect (Janiszewski & Meyvis, 2001; Zajonc, 1968): Increasing exposures to a stimulus tends to improve attitudes toward that stimulus. Second, more familiar brands were remembered better (*r* = 0.53) than less familiar brands, and more familiar logos were also better remembered (*r* = 0.33) than less familiar logos. These results corroborate the repetition effect (Hintzman, 1976; Janiszewski et al., 2003): Increasing exposures to a stimulus tend to improve recognition of that stimulus. Given that brand familiarity and logo familiarity were non-normally distributed, we also replicated these analyses with the nonparametric Spearman rank-order correlation, and the results were extremely similar, including significant positive correlations among all variables.

**Table 6:** Correlations (Pearson *r*) among variables in BRAND 2020. * *p* < .05, ** *p* < .01, *** *p* < .001

	1	2	3	4	5	6	7	8	9
1. BRAND: Brand Familiarity	—								
2. BRAND: Brand Liking	.73***	—							
3. BRAND: Brand Memorability	.53***	.42***	—						
4. BRAND: Logo Familiarity	.95***	.67***	.51***	—					
5. BRAND: Logo Liking	.69***	.60***	.37***	.72***	—				
6. BRAND: Logo Memorability	.32***	.21***	.41***	.33***	.28***	—			
7. Brand Finance: Rank (*N* = 500)	– .37***	– .22***	– .19***	– .39***	– .22***	– .11*	—		
8. Brand Finance: Value (*N* = 100)	.45***	.23*	.31**	.43***	.39***	.08	– .94***	—	
9. BrandZ: Contribution (*N* = 95)	.40***	.35***	.35***	.29**	.29**	.33**	.09	.26*	—

#### Validation (Brand Finance)

BRAND 2020 includes 500 brands, ranked by Brand Finance from best to worst in terms of brand value. To validate the scores of BRAND 2020, we examined their correlations with brand rank in Brand Finance (2019; see Table 6). Note that stronger brands have numerically low ranks (e.g., 1st), so negative correlations are expected. Brand Finance ranks brands according to an integration of their financial performance (e.g., revenues) with consumers’ “goodwill” or attitude toward the brand (i.e., brand equity). Thus, highly ranked brands should be well liked. Moreover, given that brands’ advertising expenditures are typically proportional to their financial position (Danenberg et al., 2016), highly ranked brands should also be highly familiar and memorable. Indeed, brand rank correlated negatively with brand familiarity (*r* = – 0.37), brand liking (*r* = – 0.22), and brand memorability (*r* = – 0.19).

Prior research suggests that more likable logos can improve firm performance in terms of customer loyalty and sales (Park et al., 2013). This should manifest as a correlation between logo liking and brand rank. Moreover, due to highly ranked brands’ relatively higher advertising expenditures (Danenberg et al., 2016), one might expect those brands’ logos to also be more familiar and memorable. Indeed, brand rank correlated negatively with logo familiarity (*r* = – 0.39), logo liking (*r* = – 0.22), and logo memory (*r* = – 0.11). Thus, as expected, top consumer brands (which have numerically low ranks) and their logos elicited higher familiarity, liking, and memorability than lesser consumer brands. These results support the validity of the measures in BRAND 2020.

In addition to providing brand ranks for all 500 brands, Brand Finance also provides brand values for the top 100 brands, where *brand value* is a proprietary measure combining financial performance data with brand equity measures (see Appendix). As a robustness check, we tested whether the measures in BRAND 2020 predict those brand values. To correct for substantial skew (3.38), the brand values were log-transformed, producing a more normal distribution (skew = 1.03). Note that because stronger brands have higher brand values, positive correlations are expected. Indeed, the positive correlations between the measures in BRAND 2020 and the brand values (see Table 6, line 8) mirrored the negative correlations between BRAND 2020 and brand rank (Table 6, line 7). These results support the robustness of BRAND’s predictive validity.

#### Cross-validation (BrandZ)

The brand ranks and values in the Brand Finance list, like in most other brand lists, derive from a weighting of financial performance data and brand equity measures. Because those datasets (see Table 1) are proprietary, academic researchers are not privy to how financial performance and brand equity are weighted in the brand value calculations (see Appendix for general descriptions), nor are the brand equity measures freely available to researchers. A notable exception is BrandZ, which releases a measure of *brand contribution*, which in turn is some combination of consumers’ perceptions of “meaning,” “differentiation,” and “salience.” Although academic researchers are not privy to exactly how brand contribution is measured or calculated – again, because it is proprietary – it nonetheless appears conceptually similar to the measures in BRAND. We therefore used those BrandZ contribution scores to cross-validate the measures in BRAND 2020. Of the 100 brands in BrandZ (2020), 95 were also in Brand Finance (2019), and hence BRAND 2020. As expected, the correlations with brand contribution were positive and significant (see Table 6, line 9), and they generally compared well to BRAND’s correlations with the Brand Finance ranks and values. These results further support the validity of the measures in BRAND 2020.

#### Predicting memory

As the correlations reported above reveal, familiarity predicted liking and memory (Table 6). Familiarity and liking were measured in one sample, while liking and memory were measured in a separate sample, ensuring that the same participants never assessed both familiarity and memory. We also tested, via linear regression, whether familiarity and liking collectively predicted memory. Together brand familiarity and brand liking explained 28.2% of the variance in brand memory, *F*(2, 497) = 97.43, *p* < 0.001. The two predictors were sufficiently independent, *VIF* = 2.11, but only brand familiarity significantly predicted brand memory, β = 0.48, *t* = 8.65, *p* < 0.001. Regarding the logos, together, logo familiarity and logo liking explained 11.3% of the variance in logo memory, *F*(2, 497) = 31.77, *p* < 0.001. Collinearity was again moderate but acceptable, *VIF* = 2.08, and only logo familiarity significantly predicted logo memory, β = 0.27, *t* = 4.51, *p* < 0.001. Thus, although familiarity and liking both correlated with memory, familiarity appears to be the better predictor of memory. Likability, in contrast, appears not to affect the memorability of brands or logos. This result corroborates the recent finding that the likability and the memorability of brand elements (e.g., slogans) often diverge (Hodges et al., 2024).

#### Predicting Brand Finance (2019) rank from BRAND 2020

We also tested the extent to which our BRAND measures collectively predicted brand rank. To address the unacceptably high collinearity between brand familiarity and logo familiarity (*VIF* = 13.14), we conducted two different analyses. The first analysis was a regression that included all six measures of BRAND 2020. In this analysis, we discuss the explanatory power of the BRAND measures collectively, but we refrain from interpreting the coefficients of the predictors individually (due to their collinearity). In the first block of this model, brand familiarity, brand liking, and brand memory collectively explained 14.5% of the variance in brand rank, *F*(3, 496) = 28.03, *p* < 0.001. When logo familiarity, logo liking, and logo memory were entered in a second block, they explained an additional 2.0% of the variance in brand rank, *F*(6, 493) = 16.23, *p* < 0.001. This latter result corroborates the finding that logo attitudes can affect brand equity (Park et al., 2013).

In a second analysis, we addressed collinearity by excluding logo familiarity from the model, in order to render the coefficients of the other five BRAND predictors interpretable. Collinearity among the five predictors was moderate but acceptable, all *VIF* < 3.04. Results are shown in Table 7. Importantly, only brand familiarity significantly predicted brand rank, *β* = – 0.49, *t* = – 6.79, *p* < 0.001. The unstandardized coefficient (*B* = – 36.93) indicates that for each point that a brand increases on the seven-point brand familiarity scale, it jumps an average of 37 places up the brand rankings.

**Table 7:** BRAND 2020 measures predict Brand Finance (2019) rank. ** *p* < .01, *** *p* < .001

Predictor	*B*	SE	*t*	
Constant	206.60	75.37	2.74	**
Brand Familiarity	– 36.93	5.44	– 6.79	***
Brand Liking	25.89	15.71	1.65	
Brand Memory	6.44	51.81	0.12	
Logo Liking	13.51	14.07	0.96	
Logo Memory	10.21	55.97	0.18	

*R*^2^ = 14.67%, *F*(5, 494) = 16.98, *p* < .001

In contrast, neither brand nor logo liking nor memorability significantly predicted brand rank. These findings support the common marketing assumption that brand exposure is key for consumer acceptance. How likable or memorable that exposure is appears to have little or no effect on brand performance.

#### Subgroup analysis

**Fig. 2:**
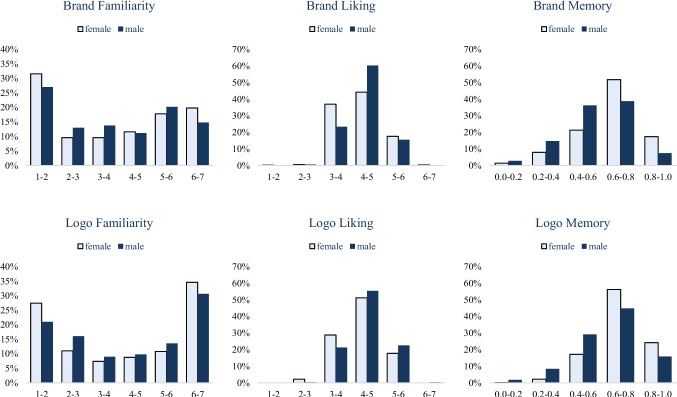
Distributions of the familiarity, liking, and memorability (corrected accuracy) of brands and their logos across genders in BRAND 2020

**Fig. 3:**
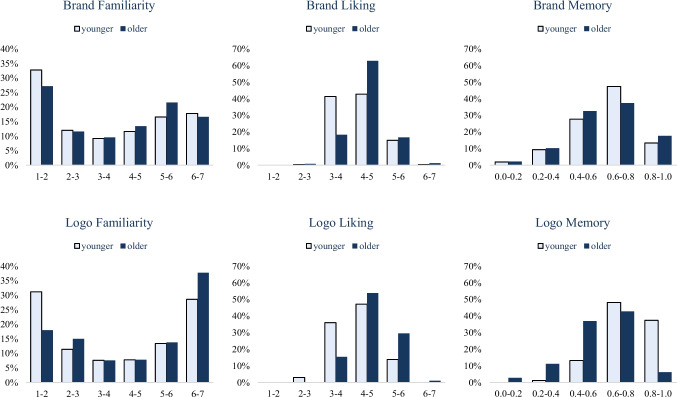
Distributions of the familiarity, liking, and memorability (corrected accuracy) of brands and their logos across ages in BRAND 2020

Finally, we also examined whether men and women, and younger and older respondents, produced similar distributions and results. As shown in Figs. 2 and 3, men and women produced similar distributions, with significant correlations between judgments by men and by women in brand familiarity (*r* = 0.91, *p* < 0.001), brand liking (*r* = 0.80, *p* < 0.001), brand memory (*r* = 0.40, *p* < 0.001), logo familiarity (*r* = 0.92, *p* < 0.001), logo liking (*r* = 0.74, *p* < 0.001), and logo memory (*r* = 0.24, *p* < 0.001).

To compare younger and older respondents, we calculated the median age of the entire sample (29.0 years), and we categorized individuals below the median as “younger” and those above as “older.” Younger and older respondents produced similar distributions of BRAND measures, with significant correlations in brand familiarity (*r* = 0.93, *p* < 0.001), brand liking (*r* = 0.79, *p* < 0.001), brand memory (*r* = 0.30, *p* < 0.001), logo familiarity (*r* = 0.92, p < 0.001), logo liking (*r* = 0.76, *p* < 0.001), and logo memory (*r* = 0.24, *p* < 0.001).

We also replicated the analysis testing whether the BRAND 2020 measures predict the Brand Finance (2019) ranks, separately for female and male respondents, and for younger and older respondents. See Tables 8, 9, 10 and 11 for results. The basic result obtained from the whole sample, whereby brand familiarity in BRAND significantly predicted Brand Finance rank (reported in the preceding section), successfully replicated within each of these four subgroups of participants.

**Table 8:** BRAND 2020 measures predict Brand Finance (2019) rank in the female subgroup. *** *p* < .001

Predictor	*B*	SE	*t*	
Constant	230.96	65.33	3.54	***
Brand Familiarity	– 32.89	5.13	– 6.41	***
Brand Liking	17.90	14.33	1.25	
Brand Memory	31.50	42.75	0.74	
Logo Liking	14.44	12.58	1.15	
Logo Memory	– 23.20	50.27	– 0.46	

*R*^2^ = 12.47%, *F*(5, 494) = 14.08, *p* < .001

**Table 9:** BRAND 2020 measures predict Brand Finance (2019) rank in the male subgroup. *** *p* < .001

Predictor	*B*	SE	*t*	
Constant	276.81	69.84	3.96	***
Brand Familiarity	– 35.13	4.69	– 7.49	***
Brand Liking	20.79	14.20	1.46	
Brand Memory	– 27.49	37.73	– 0.73	
Logo Liking	5.33	12.54	0.43	
Logo Memory	7.77	38.02	0.20	

*R*^2^ = 16.78%, *F*(5, 494) = 19.92, *p* < .001

**Table 10:** BRAND 2020 measures predict Brand Finance (2019) rank in the younger subgroup. * *p* < .05, *** *p* < .001

Predictor	*B*	SE	*t*	
Constant	155.09	68.51	2.26	*
Brand Familiarity	– 41.46	5.33	– 7.77	***
Brand Liking	27.59	14.45	1.91	
Brand Memory	57.97	39.33	1.47	
Logo Liking	20.16	11.65	1.73	
Logo Memory	12.00	51.06	0.24	

*R*^2^ = 15.89%, *F*(5, 494) = 18.67, *p* < .001

**Table 11:** BRAND 2020 measures predict Brand Finance (2019) rank in the older subgroup. *** *p* < .001

Predictor	*B*	SE	*t*	
Constant	324.00	73.15	4.43	***
Brand Familiarity	– 27.63	4.68	– 5.91	***
Brand Liking	13.84	13.74	1.01	
Brand Memory	– 20.83	37.90	– 0.55	
Logo Liking	– 3.43	12.91	– 0.27	
Logo Memory	– 0.81	38.14	– 0.02	

*R*^2^ = 12.86%, *F*(5, 494) = 14.58, *p* < .001

We conclude that the measures in BRAND 2020 are relatively stable across men and women, and across younger and older respondents. However, researchers interested in testing for more nuanced differences across respondents’ gender or age can do so with BRAND: The data file includes a pivot table function that researchers can use to specify sample characteristics, thereby allowing researchers to select specific subsamples (e.g., women aged 40–60 years) and to compare one subsample to another (for more detailed description on the pivot table, please refer to the “Data availability” section after Study 3).

### Discussion

Study 1 developed and validated BRAND 2020. Both men and women, and younger and older respondents, produced similar distributions of familiarity ratings, liking ratings, and memory scores of 500 top US brands and their logos. The liking measure in BRAND 2020 exhibited good test–retest reliability, and the correlations among measures in BRAND 2020 successfully replicated some classic effects (e.g., mere exposure). Those measures also successfully predicted the brand ranks and brand values in Brand Finance (2019), as well as the “brand contribution” in BrandZ (2020), thus supporting the predictive validity of BRAND 2020.

## Study 2: Conceptual replication of Lowrey and Shrum (2007)

Having established the validity and reliability of BRAND 2020, we next tested its utility for brand-related research by attempting to replicate an effect from the branding literature. We chose to replicate Lowrey and Shrum’s (2007) theoretically influential and practically important finding that the sounds (i.e., phonemes) in a brand name affect consumers’ brand attitudes. Specifically, Lowrey and Shrum tested the effects of front and back vowels (i.e., /i/ vs. /ɔ/, as in *Gimmel* vs. *Gommel*) on consumers’ attitudes toward brands with specific attributes (e.g., slowness or heaviness). We similarly tested whether the brands in BRAND 2020 with front versus back vowels differ in brand attitudes (i.e., brand liking in BRAND 2020).

Our study differed from Lowrey and Shrum’s (2007) original study in four key respects. First, whereas Lowrey and Shrum used only one front vowel and one back vowel (i.e., /i/ vs. /ɔ/), our analysis includes all front vowels (/i/, /ɪ/, /ɛ/, /æ/) and all back vowels (/u/, /ʌ/, /ʊ/, /ɑ/, /ɔ/), providing a more comprehensive test of the vowel effect. Second, whereas Lowrey and Shrum contrasted front and back phonemes *internal* to the name (i.e., not the first or last phoneme in the name), we instead contrast *initial* phonemes (i.e., the first phoneme in the name). This may be important because initial phonemes predict word valence better than internal phonemes (Adelman et al., 2018), and the same may be true of brand names. Third, whereas Lowrey and Shrum used fictitious brand names, our analysis instead uses real brand names, testing the vowel effect with greater ecological validity. Fourth, whereas Lowrey and Shrum examined a limited set of product categories for which specific attributes (e.g., slowness or heaviness) are important, our analysis instead includes brands from a wide variety of industries (see Table 4), thereby testing the generality of the vowel effect on brand attitudes.

### Methods

First, we selected all brand names beginning with a vowel sound (e.g., Amazon, Apple etc.) in Brand Finance (2019; *N* = 77). Then, for robustness, we retrieved phonemic transcriptions^7^ of those brand names using three different transcription programs: *Carnegie Mellon University’s Pronouncing Dictionary*, *Phon*, and *ToPhonetics*. For instance, the brand name “Amazon” is phonemically transcribed as /ˈæməˌzɑn/ and contains six different sounds (i.e., phonemes): /æ/, /m/, /ə/, /z/, /ɑ/, and /n/. Any discrepancies across the three sources of transcriptions were resolved by discussion among the authors. Next, for each brand name, we coded whether the first phoneme was a front vowel (/i/, /ɪ/, /ɛ/, or /æ/ = 1) or a back vowel (/u/, /ʌ/, /ʊ/, /ɑ/, or /ɔ/ = 0). Finally, we retrieved brand liking ratings from BRAND 2020. We used the brand names’ initial phoneme (i.e., front or back vowel) to predict their brand liking. Because both the length and the frequency of a word are associated with its affective evaluation (Adelman et al., 2018; Kuperman et al., 2014), we also included brand name length (i.e., number of phonemes) and brand familiarity (from BRAND 2020) as controls.

### Results

An independent samples *t* test revealed that brand names beginning with the back vowels /u/ (e.g., Uber), /ʌ/ (e.g., Under Armour), /ɑ/ (e.g., Optum), and /ɔ/ (e.g., Oracle) were liked more than brand names beginning with the front vowels /i/ (e.g., eBay), /ɪ/ (e.g., Instagram), /ɛ/ (e.g., Esso), and /æ/ (e.g., Amazon), (M_back_ = 4.50, SD = 0.59; M_front_ = 4.14, SD = 0.44;* t*(75) = 2.84*, p* = 0.006, Cohen’s *d* = 0.69). This result also held when adding brand name length and brand familiarity as covariates, *t*(73) = 2.37*, p* = 0.021.

### Discussion

Our result conceptually replicates and extends Lowrey and Shrum’s (2007) original finding: Brand names beginning with back vowels are liked more than brand names beginning with front vowels. Whereas Lowrey and Shrum demonstrated this effect with only a single pair of name-internal phonemes, and with only fictitious brand names in a few specific product categories, our results generalize this effect across all front and back vowels, using only the brand name’s initial phoneme, across a much larger set of real brand names from many different industries. Thus, our analysis demonstrates that the vowel effect on brand attitudes (i) is far more general than previously known, and (ii) predicts consumers’ attitudes toward real brands. By conceptually replicating and extending Lowrey and Shrum’s important finding, this study demonstrates the utility of BRAND for brand-related research.

## Study 3: BRAND 2024

An inherent limitation of BRAND 2020, or indeed of any brand ranking, is that consumers’ attitudes toward brands may change over time, yet any given set of ratings or rankings is set in time. Thus, one may question whether the brand familiarity, brand liking, and brand memory scores in BRAND 2020 remain stable across time, and hence whether BRAND 2020 will be useful for future studies. We addressed the time-limited nature of BRAND in two ways.

First, we assessed the stability of brand rankings across time. For this analysis, we compared the Brand Finance US 500 (2019) list, used in BRAND 2020, to the lists of the previous 4 years (Brand Finance, 2015, 2016, 2017, 2018) and the subsequent 4 years (Brand Finance, 2020, 2021, 2022, 2023; see Table 12). To calculate the number of overlapping brands with our target Brand Finance, 2019, we downloaded all Brand Finance reports from 2015 to 2023 and used a matching function to identify common brands. That is, for each year from 2015–2018 and 2020–2023, we counted the number of brands in common with Brand Finance (2019). However, we noticed some slight variations in the way brand names were reported in Brand Finance over the years (e.g., M.A.C. vs. MAC, Bristol-Myer Sqb vs. Bristol Myers Squibb). Although we manually corrected most of these discrepancies, we believe the number of overlapping brands might be slightly underestimated.

**Table 12:** Overlap of Brand Finance US 500 (2019) with the four preceding years (2015–2018) and the four succeeding years (2020–2023)

Report	Year	Number overlap	% Overlap
Brand Finance US 500	2015	310	62%
Brand Finance US 500	2016	370	74%
Brand Finance US 500	2017	393	79%
Brand Finance US 500	2018	444	89%
Brand Finance US 500	2019	—	—
Brand Finance US 500	2020	438	88%
Brand Finance US 500	2021	435	87%
Brand Finance US 500	2022	411	82%
Brand Finance US 500	2023	406	81%

Three hundred ten brands in Brand Finance (2019) also appeared in Brand Finance (2015), indicating that 62% of the brands in the top 500 in 2015 remained on the list 4 years later. Four hundred six of the brands from Brand Finance (2019) also appeared in the 2023 list, meaning 81% of the brands in the top 500 in 2019 remained on the list 4 years later. On average, over these 8 years, 80% of the brands in Brand Finance (2019) were consistently found in other reports, demonstrating a core group of brands that tend to make the list each year. To further enhance the longevity of our BRAND dataset, 94 new brands from the Brand Finance, 2023 list were added to BRAND 2024.

The second and more direct way that we assessed the time-sensitive nature of BRAND 2020 was by replicating it 4 years later (i.e., BRAND 2024). For BRAND 2024, we included all brands that appeared in BRAND 2020 (except a handful that ceased operating between 2020 and 2024), as well as all brands that appeared in the Brand Finance (2023) US 500. Thus, BRAND 2024 allowed us to test the stability of brand attitudes across a 4-year period, while also providing a more current list of top brands in 2024. Note that in BRAND 2024 we collected familiarity and liking ratings of these top brands and their logos, but we did not include the memory measures of BRAND 2020, due to their more intensive procedures and less reliable results. In total, BRAND 2024 includes familiarity and liking ratings of 589 top brands and their logos, for a total of 2356 aggregated datapoints (i.e., 589 brands and 589 logos × 2 measures). Those 2356 datapoints are aggregated from approximately 94,000 responses from approximately 800 US-resident consumers. Each respondent evaluated 59 brands or logos, and each datapoint is aggregated across approximately 40 consumer responses.

### Methods

#### Participants

Eight hundred three respondents from the Prolific online research panel participated for pay. Respondents’ ages ranged from 18 to 83, with a mean of 41 years (SD = 14). 53.7% self-identified as female, 43.8% as male, 1.9% as “other,” and 0.6% chose not to say. As in BRAND 2020, we used a screening function in Prolific to restrict eligibility to users who reported current residence in the US.

Data were collected from two independent groups sequentially. As in BRAND 2020, each participant judged either the brands or their logos, but not both. Of the 803 US-resident participants, approximately half (*N* = 402) rated their familiarity with and liking of the brands, and after excluding two participants who failed an attention check (see Study 1), 400 valid participants remained in this group. The other half of the participants (*N* = 401) instead rated their familiarity with and liking of the logos, and after excluding one participant who failed the attention check, 400 valid participants remained in this group. Table 13 summarizes basic demographic characteristics of the respondents in each group.

**Table 13:** Respondent characteristics (after exclusions) in BRAND 2024

			Age				Gender	
Group	Stimuli	*N*	Min	Max	M	SD	Female	Male
Familiarity-Liking	Brands	400	18	78	41.43	14.16	60%	40%
	Logos	400	18	83	41.38	13.32	50%	50%
	Overall	800	18	83	41.41	13.74	55%	45%

#### Stimuli

In developing BRAND 2024, we sought to include (i) all 500 brands from BRAND 2020 that remained operational in 2024 (i.e., excluding all brands that went out of business by 2024), and (ii) all 500 brands from Brand Finance (2023). Thus, we first checked whether each of the brands in BRAND 2020 was still operational in 2024. Of those 500 brands, 492 (i.e., 98%) were still operational in 2024. Eight brands in BRAND 2020 (i.e., BB&T, SunTrust Bank, Twitter, Schlumberger, Waste Management, Kraft, Heinz, and L3) ceased operating by 2024, as they underwent either a renaming (i.e., Twitter, Schlumberger, Waste Management, and L3 respectively became X, SLB, WM, and L3 Harris) or a merger (i.e., BB&T and SunTrust Bank merged into Truist, and Kraft and Heinz merged into Kraft Heinz). In BRAND 2024, we did not include these eight non-existing brands. Thus, 492 brands in BRAND 2020 also appear in BRAND 2024.

In addition to those 492 brands from BRAND 2020, BRAND 2024 also included 97 new brands. These included 91 new brands that appeared in the Brand Finance (2023) list and the six brands of the mergers/rebrands. Thus, BRAND 2024 included 589 brands and their logos. To simplify data collection, we included a filler non-US brand (Pret a Manger) so that we were able to create ten lists of 59 brands each. In this way, all participants rated the same number of brands. However, this brand was not included in the final analysis or dataset.

For each of the 492 brands that appeared in both BRAND 2020 and BRAND 2024, we checked whether the brand still used in 2024 the same logo that was included in BRAND 2020. Only 62 brands (13%) updated their logo between 2020 and 2024. For BRAND 2024, we used each brand’s current logo in 2024 (i.e., for 62 brands, their logos differ in BRAND 2020 and BRAND 2024). We also collected the logos of the 97 new brands (+ the 1 filler brand) following the same selection criteria as in BRAND 2020 (see Table 4). Finally, as in BRAND 2020, we resized all logos to have similar dimensions within a standardized frame, and we saved them as 400 × 225 resolution png files.

#### Procedure

The procedure was identical to the familiarity-liking group of BRAND 2020, except that here each of the ten experimental lists included 59 brands or logos (instead of 50), and hence each participant rated their familiarity and liking of 59 stimuli.^8^

### Results

#### Data aggregation

The data of BRAND 2024 were aggregated in the same way as BRAND 2020, with the brand as the unit of analysis. Each brand was rated for familiarity and liking by approximately 40 participants (min = 39, max = 41, SD = 0.77), and each logo received approximately 40 familiarity and liking ratings (min = 37, max = 41, SD = 1.10).

**Fig. 4:**
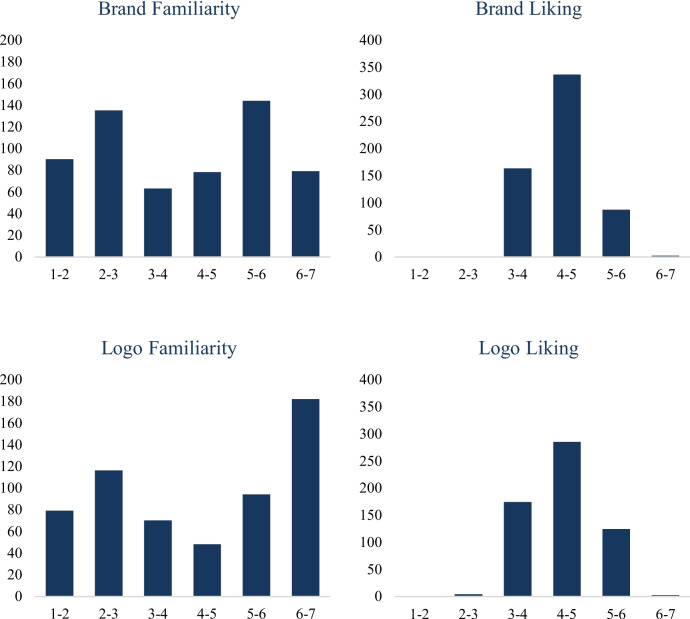
Distributions of the familiarity and liking of brands and their logos in BRAND 2024

*Distributions.* Descriptive statistics are reported in Table 14, and histograms of the four measures are shown in Fig. 4. *Brand familiarity* ratings ranged from 1.27 (S-26) to 6.88 (YouTube), with a mean of 3.97 (SD = 1.69). Notably, however, the distribution was bimodal (Fig. 4). *Brand liking* ratings ranged from 2.88 (Newport) to 6.12 (YouTube), with a mean of 4.36 (SD = 0.54). As shown in Fig. 4, the distribution was normal. *Logo familiarity* ratings ranged from 1.43 (Arrow Electronics) to 6.97 (Microsoft), with a mean of 4.36 (SD = 1.85). As with brands, logo familiarity shows a bimodal distribution with peaks near 1 and 7 (Fig. 4). *Logo liking* ratings ranged from 2.48 (Arrow Electronics) to 6.12 (Google), with a mean of 4.39 (SD = 0.67) and were normally distributed.

**Table 14:** Descriptive statistics of measured variables in BRAND 2024

Stimuli	Measure	*N*	Min	Max	M	SD	Skew
Brands	Familiarity	589	1.27	6.88	3.97	1.69	0.02
	Liking	589	2.88	6.12	4.36	0.54	0.69
Logos	Familiarity	589	1.43	6.97	4.36	1.85	– 0.13
	Liking	589	2.48	6.12	4.39	0.67	0.15

#### Correlations among measures

Table 15 displays the correlations among the measures of BRAND 2024 (see lines 1-4). As expected, the four measures all correlated positively with each other. As in BRAND 2020, more familiar brands were liked more (*r* = 0.74) than less familiar brands, and more familiar logos were also liked more (*r* = 0.78) than less familiar logos, again corroborating the mere exposure effect (Janiszewski & Meyvis, 2001; Zajonc, 1968).

**Table 15:** Correlations (Pearson *r*) among variables in BRAND 2024. * *p* < .05, ** *p* < .01, *** *p* < .001

	1	2	3	4	5	6
1. BRAND: Brand Familiarity	—					
2. BRAND: Brand Liking	.74***	—				
3. BRAND: Logo Familiarity	.95***	.68***	—			
4. BRAND: Logo Liking	.74***	.62***	.78***	—		
5. Brand Finance: Rank (*N* = 499)	– .37***	– .22***	– .37***	– .26***	—	
6. Brand Finance: Value (*N* = 100)	.41***	.23*	.32**	.34***	– .71***	—

#### Validation (Brand Finance)

To validate the scores of BRAND 2024, we examined their correlations with brand rank in Brand Finance (2023; see Table 15).^9^ Given that stronger brands have numerically low ranks (e.g., 1st), negative correlations are expected. As in BRAND 2020, brand rank correlated negatively with both brand familiarity (*r* = – 0.37) and brand liking (*r* = – 0.22) in BRAND 2024. Also as in BRAND 2020, brand rank correlated negatively with logo familiarity (*r* = – 0.37) and logo liking (*r* = – 0.26). These results support the validity of the measures in BRAND 2024.

Brand Finance (2023) also provides brand values for the top 100 brands, where *brand value* combines financial performance data with brand equity measures (see Appendix). As a robustness check, we tested whether the measures in BRAND 2024 predict those brand values. To correct for substantial skew (4.13), the brand values were log-transformed, producing a more normal distribution (skew = 1.26). Given that stronger brands have higher brand values, positive correlations are expected. Indeed, the positive correlations between the measures in BRAND 2024 and the brand values (see Table 15, line 6) mirrored the negative correlations between BRAND 2024 and brand rank (Table 15, line 5). These results support the robustness of BRAND 2024’s predictive validity.

#### Predicting Brand Finance (2023) rank from BRAND 2024

We also tested whether BRAND 2024 predicted brand rank. To address the unacceptably high collinearity between brand familiarity and logo familiarity (*VIF* = 12.27), we first conducted a regression that included all four measures of BRAND 2024 (brand familiarity, brand liking, logo familiarity, and logo liking), but we refrain from interpreting the coefficients of the predictors individually (due to their collinearity). In the first block of this model, brand familiarity and brand liking collectively explained 14.5% of the variance in brand rank, *F*(2, 496) = 41.85, *p* < 0.001. When logo familiarity and logo liking were entered in a second block, they explained an additional 0.6% of the variance in brand rank, *F*(4, 494) = 21.93, *p* < 0.01. In a second analysis, we addressed collinearity by excluding logo familiarity from the model, and collinearity among the three predictors was moderate but acceptable (all *VIF* < 3.06). Results are shown in Table 16. Brand familiarity significantly predicted brand rank, *β* = – 0.47, *t* = – 6.55, *p* < 0.001, and brand liking marginally predicted brand rank, *β* = – 0.10, *t* = 1.65, *p* < 0.10. The unstandardized coefficient (*B* = – 40.46) indicates that for each point that a brand increases on the seven-point brand familiarity scale, it jumps an average of 40 places up the brand rankings.

**Table 16:** BRAND 2024 measures predict Brand Finance (2023) rank. *** *p* < .001

Predictor	*B*	SE	*t*	
Constant	261.16	65.97	3.96	***
Brand Familiarity	– 40.46	6.18	– 6.55	***
Brand Liking	26.88	16.27	1.65	
Logo Liking	8.22	13.67	0.60	

*R*^2^ = 14.50%, *F*(3, 495) = 27.98, *p* < .001

#### Subgroup analysis

**Fig. 5:**
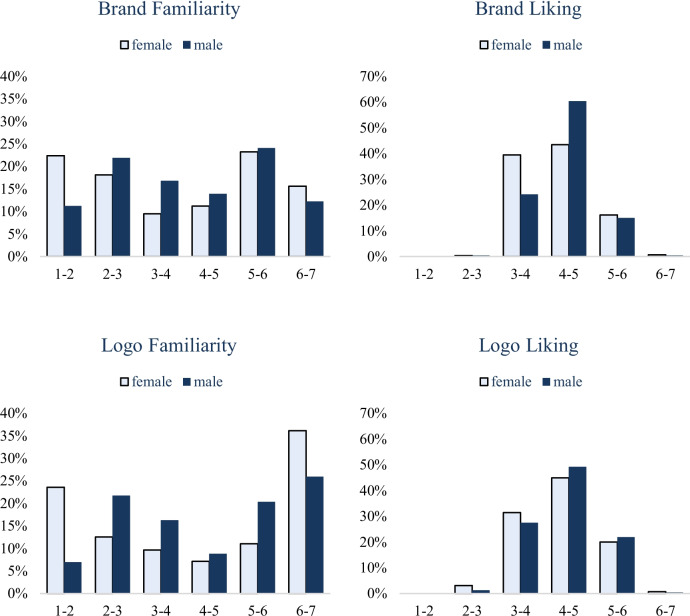
Distributions of the familiarity and liking of brands and their logos across genders in BRAND 2024

**Fig. 6:**
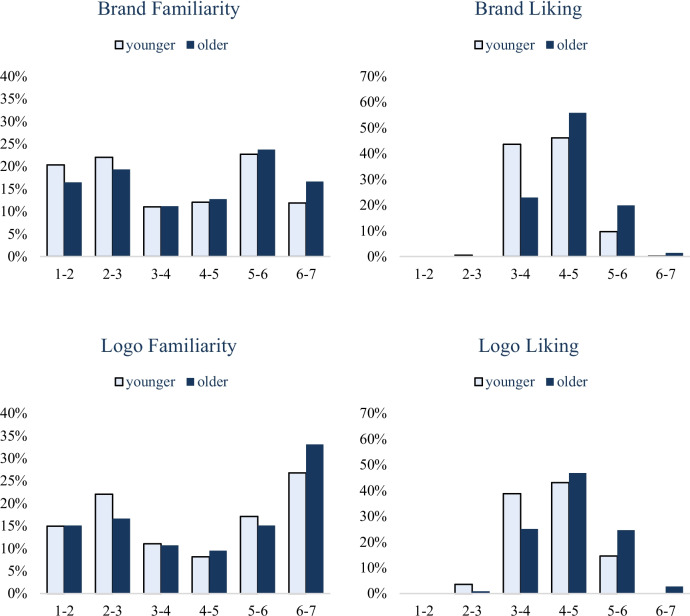
Distributions of the familiarity and liking of brands and their logos across ages in BRAND 2024

Figures 5 and 6 show the four measures of BRAND 2024 by men and women separately, and by younger and older respondents separately, with strongly convergent distributions. Men and women rated brand familiarity (*r* = 0.90, *p* < 0.001), logo familiarity (*r* = 0.93, *p* < 0.001), brand liking (*r* = 0.66, *p* < 0.001) and logo liking (*r* = 0.78, *p* < 0.001) highly similarly. For the comparison of younger and older respondents, we again calculated the median age of the entire sample (38.0 years), categorizing individuals below the median as younger and those above as older. As in BRAND 2020, here in BRAND 2024 younger and older respondents provided highly similar patterns of ratings for brand familiarity (*r* = 0.93, *p* < 0.001), brand liking (*r* = 0.74, *p* < 0.001), logo familiarity (*r* = 0.93, *p* < 0.001), and logo liking (*r* = 0.72, *p* < 0.001). We also tested whether the BRAND 2024 measures predict the Brand Finance (2023) ranks, separately for female and male respondents and for younger and older respondents (see Tables 17, 18, 19 and 20). As found with the whole sample (see the preceding section), brand familiarity in BRAND significantly predicted Brand Finance rank within each of these four subgroups of participants.

**Table 17:** BRAND 2024 measures predict Brand Finance (2023) rank in the female subgroup. *** *p* < .001

Predictor	*B*	SE	*t*	
Constant	234.05	54.74	4.28	***
Brand Familiarity	– 40.51	5.59	– 7.25	***
Brand Liking	25.60	14.21	1.80	
Logo Liking	15.68	11.69	1.34	

*R*^*2*^ = 14.50%, *F*(3, 495) = 27.98, *p* < .001

**Table 18:** BRAND 2024 measures predict Brand Finance (2023) rank in the male subgroup. *** *p* < .001

Predictor	*B*	SE	*t*	
Constant	402.97	64.26	6.27	***
Brand Familiarity	– 31.62	5.97	– 5.30	***
Brand Liking	5.11	14.16	0.36	
Logo Liking	– 9.81	11.78	– 0.83	

*R*^*2*^ = 13.36%, *F*(3, 495) = 25.45, *p* < .001

**Table 19:** BRAND 2024 measures predict Brand Finance (2023) rank in the younger subgroup. *** *p* < .001

Predictor	*B*	SE	*t*	
Constant	290.32	58.99	4.92	***
Brand Familiarity	– 36.96	5.71	– 6.48	***
Brand Liking	20.11	14.57	1.38	
Logo Liking	4.63	12.73	0.36	

*R*^*2*^ = 14.17%, *F*(3, 495) = 27.24, *p* < .001

**Table 20:** BRAND 2024 measures predict Brand Finance (2023) rank in the older subgroup. *** *p* < .001

Predictor	*B*	SE	*t*	
Constant	298.51	60.04	4.97	***
Brand Familiarity	– 35.67	5.64	– 6.32	***
Brand Liking	21.23	14.11	1.50	
Logo Liking	1.47	10.84	0.14	

*R*^*2*^ = 13.34%, *F*(3, 495) = 25.4, *p* < .001

#### Predicting Brand Finance (2023) rank from BRAND 2020

To analyze the robustness of the BRAND dataset across time, we tested the extent to which our BRAND 2020 measures (familiarity and liking) predict the 2023 Brand Finance rank. We first conducted a regression that included four measures of BRAND 2020 (brand familiarity, brand liking, logo familiarity, and logo liking). In the first block of this model, brand familiarity and brand liking collectively explained 11.65% of the variance in brand rank, *F*(2, 399) = 26.32, *p* < 0.001. When logo familiarity and logo liking were entered in a second block, they explained an additional 1.58% of the variance in brand rank, *F*(4, 397) = 15.14, *p* < 0.01. Thus, BRAND 2020 predicted brand rank in 2023.

In a second analysis, we addressed the unacceptably high collinearity between brand familiarity and logo familiarity (*VIF* = 11.99) by excluding logo familiarity from the model. Collinearity among the three remaining predictors was moderate but acceptable, all *VIF* < 2.66. Results are shown in Table 21. Only brand familiarity significantly predicted brand rank, *β* = – 0.39, *t* = – 5.01, *p* < 0.001. The unstandardized coefficient (*B* = – 28.00) indicates that for each point that a brand increases on the seven-point brand familiarity scale, it jumps an average of 28 places up the brand rankings. In contrast, neither brand nor logo liking significantly predicted brand rank. These findings support the robustness of the BRAND dataset across time.

**Table 21:** BRAND 2020 measures predict Brand Finance (2023) rank. *** *p* < .001

Predictor	*B*	SE	*t*	
Constant	269.97	72.08	3.75	***
Brand Familiarity	– 28.00	5.59	– 5.01	***
Brand Liking	2.58	16.31	0.16	
Logo Liking	11.52	16.00	0.72	

*R*^2^ = 11.77%, *F*(3, 398) = 17.70, *p* < .001

#### Predicting change in Brand Finance rank

Finally, we also investigated whether changes between BRAND 2020 and BRAND 2024 predict changes in Brand Finance rank between 2019 and 2023. Results are shown in Table 22. Improvements in brand rank were associated with increases in brand familiarity (*r* = – 0.114, *p* = 0.023) but not with changes in brand liking (*r* = – 0.069, *p* = 0.165), logo familiarity (*r* = – 0.064, *p* = 0.203), or logo liking (*r* = – 0.053, *p* = 0.295). These results indicate that changes in our participants’ brand familiarity in BRAND track changes in Brand Finance’s brand ranks.

**Table 22:** Change in BRAND measures predicts change in Brand Finance rank. * *p* < .05, ** *p* < .01

Predictor	*B*	SE	*t*	
Constant	6.49	4.12	1.58	
Δ Brand Familiarity	– 19.14	6.90	– 2.77	**
Δ Brand Liking	24.50	11.42	2.14	*
Δ Logo Liking	10.55	9.33	1.13	

*R*^2^ = 2.68%, *F*(3, 398) = 3.65, *p* = .01

### Discussion

BRAND 2024 serves two primary purposes. First, it provides a more current database of consumers’ familiarity with and liking of more than 500 top US brands. Specifically, it includes all 492 surviving brands from BRAND 2020 (i.e., excluding eight brands that ceased trading between 2020 and 2024), plus 97 new entrants that were not present on the Brand Finance (2019) list, for a total of 589 brands. Second, BRAND 2024 also provided evidence of BRAND's reliability, validity, and utility across several years. The distributions of familiarity and liking ratings in BRAND 2024 were highly similar to those of BRAND 2020, as were the correlations among the measures. As with BRAND 2020, the measures in BRAND 2024 predicted Brand Finance’s brand ranks and brand values. Perhaps most importantly, BRAND 2020 successfully predicted Brand Finance (2023) rank. That is, the measures of BRAND 2020 retained good predictive validity 4 years later. Finally, we also found that changes between BRAND 2020 and BRAND 2024 predicted changes in Brand Finance rank from 2020 to 2024. Collectively, these results suggest that BRAND will be useful for future studies, despite the time-specific nature of the measures.

## Data availability

We provide two files that foster easy, user-friendly access to the BRAND dataset (accessible at https://researchbox.org/1892).

### The BRAND dataset

The main “BRAND Dataset” file contains all the summary measures from BRAND 2020, BRAND 2024, and various external reports on brand value. Each of the 597 brands that we studied occupies a single row. Alongside the brand name is the product category, the logo used in BRAND 2020, and the logo used in BRAND 2024. The remaining columns are split between BRAND 2020, BRAND 2024, and the independent reports (e.g., Brand Finance). Within both BRAND 2020 and BRAND 2024 are the measures of brand familiarity, brand liking, logo familiarity, and logo liking; for each there is the number of respondents, the mean, and the standard deviation. In BRAND 2020 only, there are summaries of the recognition memory data for brands and logos. For each brand and logo, the number of respondents, proportion of hits (correct answers to seen brands), the proportion of misses (incorrect answers to seen brands), the proportion of correct rejections (correct answers to unseen brands), the proportion of false alarms (incorrect answers to unseen brands) and corrected accuracy (hits minus false alarms) are given. Signal detection measures are also computed: *d’* or sensitivity – a measure of how distinct the representation of the brand is when it has or has not been seen – and criterion – a measure of whether decisions are biased towards responding either that a brand has been seen or that it has not, with values above zero indicated a bias towards “new” responses (more correct rejections than hits). In the independent reports, brand rankings and value where available are listed from Brand Finance, 2019, Brand Finance, 2023, BrandZ, 2020, InterBrand, 2020, Forbes, 2020, and Tenet, 2020. BrandZ additionally measures “brand contribution,” and Forbes measures “brand revenue,” whereas Tenet only provides brand rank.

### The BRAND pivot table

The data are also provided in the “BRAND PivotTable” file in a format that enables researchers to explore subsets of participants based on criteria other than those we have reported in this article. In the first tab of this file, there is a table in which for each brand there is the average brand familiarity, average brand liking, and brand *d*’, and the average logo familiarity, average logo liking, and logo *d*’. By default, these are computed on all the data, so that familiarity and liking averages contain data from both BRAND 2020 and BRAND 2024 participants. Above this table, there are filters that can be applied to restrict the data to various subgroups based on age and gender, whether they are from the 2020 or 2024 data set, and whether they completed the familiarity or memory task. This would allow researchers to, for instance, investigate the similarity of ratings across three different age groups rather than the median split we have considered or to construct control measures for a specific demographic subgroup, such as females under 30. The second tab of this file contains the underlying data with one row for each combination of subject and brand that occurred.

## General discussion

BRAND is the most comprehensive freely available dataset of frequently researched consumer responses to branding stimuli, with measures of familiarity (awareness), liking (attitudes), and memory (recognition) of more than 500 top brands and their logos, spanning 32 industries. Overall, BRAND includes 5356 primary datapoints aggregated from 244,400 raw datapoints (i.e., individual familiarity, liking, and memory responses) collected from 2000 US-resident consumers in two different years (i.e., 2020 and 2024).

To generate and validate BRAND, we conducted three studies. Study 1 was conducted in 2020 on the Brand Finance (2019) US 500 list and generated BRAND 2020. It contains 3000 primary datapoints (500 brands + 500 logos × 3 measures) aggregated from 150,000 raw datapoints (i.e., individual familiarity, liking, and memory responses) collected from 1200 US-resident consumers. In this study, the data exhibit good reliability, as shown by a strong convergence of ratings by independent groups of participants; face validity, as shown by intuitively predicted intercorrelations among variables; external validity, as shown by replications of well-known relationships among variables; robustness, as shown by replications of those relationships across multiple measures from the same source and across different subgroups (i.e., males and females, younger and older adults); cross-validity, as shown by replications of those relationships in a different source; and discriminant validity, as shown by the measures’ unique contributions to predicting brand rank.

Study 2 tested the external validity and utility of BRAND by replicating and generalizing a prior result from the branding literature. Specifically, using fictitious brand names, Lowrey and Shrum (2007) found that consumers like brand names with back vowels (e.g., “Nallen”) more than those with front vowels (e.g., “Nillen”). Study 2 reveals that this effect also holds across more front and back vowels and a larger set of real brands in BRAND 2020, demonstrating the validity and utility of BRAND.

Finally, Study 3 generated and validated BRAND 2024. Conducted in 2024 on the Brand Finance (2023) US 500 list, BRAND 2024 includes 2356 primary datapoints (589 brands + 589 logos × 2 measures) aggregated from 94,400 raw datapoints (i.e., individual familiarity and liking responses) collected from 800 US-resident consumers. Validity checks are consistent with those of Study 1: The data exhibit good face validity, as shown by intuitively predicted intercorrelations among variables; external validity, as shown by replications of well-known relationships among variables; robustness, as shown by replications of those relationships across different subgroups (i.e., males and females, younger and older adults) and across time; and discriminant validity, as shown by the measures’ unique contributions to predicting brand rank. Additionally, this study also shows that the measures of BRAND 2020 retained good predictive validity 4 years later, and that changes between BRAND 2020 and BRAND 2024 predicted changes in Brand Finance rank from 2020 to 2024. In sum, these three studies demonstrate the reliability, validity, and utility of BRAND across respondent samples and several years.

BRAND can facilitate research not only in consumer behavior and psychology but also in several related academic disciplines (e.g., economics, management, marketing). It can be broadly useful for testing hypotheses involving branding stimuli or consumer responses to brands, and for selecting stimuli in brand-related research. Of course, BRAND also has important limitations. Below we consider a few of those limitations before concluding with some future directions.

## Limitations

In creating BRAND, we faced three fundamental decisions, each of which averted some limitations but introduced others. The first of those fundamental decisions was which brands to include in BRAND. Our primary goal was to maximize the utility of BRAND as a research tool. Consequently, we sought as large a list of brands as we could feasibly measure. As shown in Table 1, the Brand Finance list was by far the most extensive preexisting list, so we chose it as the basis for BRAND. For better or worse, however, the Brand Finance list includes only “US brands.” While the US currently is the locale for much of the published research on brands, and hence the restriction to US brands delivers ample utility in its own right, we nevertheless consider this regional restriction to be an important limitation of BRAND. On the other hand, we do note that by virtue of including the top “US brands,” BRAND also includes about half of the world’s top “global brands.” We thus believe that BRAND can also be useful for research conducted beyond the US market.

A second fundamental decision was which brand element(s) to include. Most branding research examines brands in general. For instance, research on brand attitudes typically does not specify what particular aspect of the brand is evaluated; researchers simply ask consumers to evaluate the given brand. We therefore included such general measures of brand awareness, attitudes, and recognition in BRAND, as these are key indicators of brand equity (Keller, 1993). However, to amplify the utility of BRAND for researchers, we also sought to include measures of a more specific brand element, such as the brand name or logo. Although we believe that evaluations of the brand name per se could be interesting, we suspect that evaluations of the *brand* and of the *brand name* would diverge only minimally. For instance, if one is familiar with the Nike brand, they must also be familiar with the Nike brand name. Furthermore, if Nike (the brand) is highly memorable, we suspect that Nike (the brand name) would be similarly memorable. Thus, we opted not to include measures of the brand name per se in BRAND. In contrast, logos seemed more likely to yield at least somewhat divergent data. For instance, BRAND reveals that although consumers do not particularly like Emerson Electric, they do like the Emerson Electric logo. And conversely, consumers generally like Dollar General, though they do not particularly like the Dollar General logo. Thus, we chose to include brands (in general) and their logos (in particular) in BRAND, resulting in the omission of other important brand elements (e.g., brand names).

The third and final fundamental decision was which measure(s) to include. We viewed familiarity, which may be considered a proxy for brand or logo awareness, as perhaps the most broadly useful measure that a branding database could have, as testified by the already existing abundant work on the topic (e.g., Campbell & Keller, 2003; Kent & Allen, 1994; Percy & Rossiter, 1992). Indeed, familiarity (or awareness) can serve as either a predictor or a control factor in a great variety of studies. We also measured liking, which is a proxy for brand or logo attitudes, because it is among the most common dependent variables in branding and consumer research (e.g., Keller, 1987, 1993; Percy & Rossiter, 1992). Given the relevance of awareness and attitudes, we collected such ratings for both brands and logos in different years, both 2020 and 2024. Lastly, we included memorability because we suspect that interest among researchers exceeds the number of published studies on brand and logo memorability (e.g., Keller, 1987). That is, we believe that the relative difficulty of conducting memory tests has hindered the progress of research on this important topic, and we hope that the memory measures in BRAND 2020 will facilitate substantial advances in the understanding of brand and logo memorability. Once again, however, our decision to measure these three key indicators of brand equity (i.e., familiarity, liking, and memory; Keller, 1993) unfortunately led us to exclude other important and useful measures, such as purchase likelihood and brand loyalty. As explained next, the limited number of measures in BRAND also provides clear opportunities for further research.

## Future directions

We believe that BRAND can provide even more contributions than it currently does because BRAND is inherently incomplete. As explained above, our choices of brands, brand elements, and measures introduced limitations. Fortunately, however, BRAND is also inherently expandable. We envision BRAND as a living database.

To illustrate, in the field of psycholinguistics, there are standard datasets that hundreds of independent researchers use for their own research purposes. For instance, Balota et al. (2007) created the English Lexicon Project (*ELP*), which measured word recognition times (i.e., reading aloud and lexical decisions) for virtually every word in the English language. Subsequently, other researchers published supplementary datasets. For example, Warriner et al. (2013) added measures of emotional arousal and valence for many of the words in the ELP. From this collaborative effort emerged a research subfield in which novel hypotheses are tested with standardized measures and within standard datasets, often without the collection of a single new datapoint. For instance, Kuperman et al. (2014) simply merged the datasets of Warriner et al. and Balota et al. to test the effects of arousal and valence on word recognition. Similarly, Adelman and Estes (2013) used arousal and valence ratings to predict the recognition memory scores collected by Cortese et al. (2010). Adelman et al. (2018) used Warriner et al.’s emotion norms to identify phonemes with positive or negative associations, and then used those emotional associations to predict the reading aloud times in the ELP. More generally, any researcher investigating language processing can use those and other datasets to test hypotheses and/or to select word stimuli that are matched or varied on many specific properties (e.g., frequency, concreteness).

Similarly, we envision BRAND as a foundation for expansion. It can be expanded to include a more comprehensive list of global brands, rather than only US-based brands. It can be expanded to include measures of the brand name, or other brand elements such as slogans. It can also be expanded to include additional measures, such as brand commitment and purchase likelihood. With open research practices such as making data freely available, as we have done here, BRAND can easily be accessed, expanded, and merged with other datasets (e.g., Compustat) to facilitate research on a very broad range of topics within and beyond consumer behavior.

## Data Availability

The data and materials are available at the same link from the Open Practices statement.

## Appendix: Brand valuation methods

**Brand Finance:** Brand Finance calculates brand value using the Royalty Relief approach. First, it derives a *Brand Strength Index* (BSI) incorporating marketing investment, stakeholder equity, and business performance. Second, it sets a *royalty range* for the specific industry based on the licensing agreements within that field. Third, it combines the BSI and royalty range to determine a *royalty rate*, which estimates the percentage of total revenues that can be attributed to the brand. Next, it determines *forecast revenues* by combining data from historic revenues, equity analyst forecasts, and economic growth rates. The royalty rate is then applied to the forecasted revenues to derive *brand revenues*. Lastly, *brand value* is attained by discounting post-tax brand revenues to a net present value.

**BrandZ:** BrandZ first determines the *attribution rate*, which is the percentage of corporate earnings pertaining to the brand. Next, corporate earnings are multiplied by the attribution rate, resulting in the *brand multiple*. Then the earnings of the brand are multiplied by the brand multiple to obtain the *financial value*. However, BrandZ also emphasizes the intangible assets of the brand itself, consisting of three components: being *meaningful* (emotional and rational affinity), being *different* (from a consumer perspective) and being *salient* (easy to recall). These three components yield the *brand contribution*, which is the purchase volume and price premium that can be attributed to the brand. Finally, the financial value is multiplied by brand contribution, resulting in *brand value*.

**Forbes:** Forbes first determines the revenues and earnings before interest and taxes (*EBIT*). It then takes the average of the last 3 years’ EBIT and discounts a change of 8% of the brand’s capital employed (i.e., Forbes assumes that a generic brand should earn at least 8% on this capital). Next, a proportion of those earnings are allocated to the brand, considering the influence of brands within the given industry. Lastly, it applies the average price to earnings multiple over the past 3 years to net brand earnings, yielding *brand value*.

**Interbrand:** *Brand value* consists of three main components: a performance analysis of the financials, the role played by brands in purchase decisions, and the brand’s competitive strength. Financial data are gathered from Refinitiv, company annual reports, investor presentations, and analyst reports. Social media data are based on text analytics and social listening by Infegy. Lastly, consumer goods data, specifically brand volumes and values, are from GlobalData.

**Tenet:** *Brand power* (cf. brand value) is derived from a CoreBrand Index, which is an extensive research exercise that includes all S&P 500 companies, across 50 industries. Approximately 10,000 consumers and brand decision-makers take part in the research, which measures brand awareness (familiarity) and brand perception (favorability) for each company. These two metrics are then combined into brand strength. Notably, brand power does not take the brands’ financial performance into account.

## Footnotes

1 The datasets of Study 1 are anonymized and cleaned from incomplete responses and responses with the same response ID. Age, sex, and residence data for the first two surveys (brand and logo familiarity-liking) were retrieved from the Prolific platform and added after initial data collection due to a technical issue. For Study 1, sex and gender were merged and labeled as “gender” for simplicity.

2 The four groups were administered sequentially, in the following order: brand familiarity and liking, logo familiarity and liking, brand liking and memory, logo liking and memory. We present them as a single study for simplicity.

3 Two entries in the Brand Finance (2019) list each included two brand names: Tide/Ariel and Stop & Shop/Giant. We included in BRAND the brands with the strongest US presence: Tide and Stop & Shop.

4 In the brand *familiarity-liking* survey only, an additional text entry (i.e., line 51) was automatically generated at the end of each list, and participants rated familiarity with and liking of a blank cell. Those ratings were not included in the analysis given their lack of validity.

5 Note that participants who reported that all stimuli appeared are not included in this mean estimate. If those participants are included (with an estimate of 0), then the mean estimate of missed logos approached zero.

6 These sample sizes (i.e., number of ratings per stimulus) are relatively large for a dataset of this type. Common rules of thumb for numbers of participants rating lexical characteristics of words, for instance, are 10 (e.g., Perry et al., 2015; Winter et al., 2024), 20 (e.g., Heyman et al., 2016; Kuperman et al., 2012; Stadthagen-Gonzalez & Davis, 2006; Su et al., 2023; Warriner et al., 2013; Xu et al., 2022), and 30 (e.g., Balota et al., 2001; Cortese et al., 2015). The sample sizes in BRAND meet or exceed all of those rules of thumb.

7 A phonemic transcription is the visual representation of speech sounds by means of symbols. The most common type of phonemic transcription uses an internationally and cross-linguistically recognized alphabet called International Phonetic Alphabet (IPA), in which one sound corresponds to one symbol.

8 As in the liking-memory condition of BRAND 2020, at the end of the study, participants were asked whether any of the stimuli did not appear on their screen. Only seven participants indicated that some logos did not appear, and those participants’ mean estimate of the number of stimuli that did not appear was 2. No participant estimated having missed more than ten logos, so following the criterion of BRAND 2020, those participants’ data were retained in the BRAND 2024 analyses.

9 Brand Finance (2023) included both Heinz and Kraft Heinz. Because Kraft Heinz was a merger of Kraft and Heinz, we included Kraft Heinz in BRAND 2024, but we did not include Heinz alone. Thus, this analysis included 499 brands rather than 500.

## Code availability

The R code is available at the same link from the Open Practices statement.

## Declarations

### Open practices statement

All materials, data, and analytic scripts are publicly available in the ResearchBox repository https://researchbox.org/1892. None of the reported studies were preregistered.
